# Laparoscopic Hiatal Repair and Gastropexy without Fundoplication of Hiatal Hernia in Elderly Patients with High Dysphagia Score: A Case Series of Four Patients

**DOI:** 10.70352/scrj.cr.25-0235

**Published:** 2025-06-12

**Authors:** Shinya Urakawa, Daishi Yoshimura, Kazuya Sakata, Kimimasa Ikeda, Satoru Miyazaki

**Affiliations:** 1 Department of Gastroenterological Surgery, Osaka Habikino Medical Center, Habikino, Osaka, Japan; 2 Department of Gastroenterological Surgery, Minoh City Hospital, Minoh, Osaka, Japan

**Keywords:** hiatal hernia, elderly patients, high dysphagia scores, hiatal repair and gastropexy

## Abstract

**INTRODUCTION:**

Hiatal repair with fundoplication for hiatal hernia (HH) could cause postoperative dysphagia. In the aging society, the number of patients with HH type III/IV and severe dysphagia is increasing. In this case, the surgical priority is to resolve the dysphagia. Recent papers have reported that laparoscopic hiatal repair without fundoplication can be an alternative procedure. Nevertheless, the indication for hiatal repair without fundoplication should be carefully considered.

**CASE PRESENTATION:**

We performed laparoscopic hiatal repair and gastropexy without fundoplication in four patients with HH type III and high dysphagia scores. The median age was 84 (range 81–96) years, and the median values in the updated Charlson Comorbidity Index (uCCI) were 2 (2–5). Dysphagia scores were high (3, n = 2 and 4, n = 2). The media operative time was 196 (57–249) minutes, and the postoperative hospital stay was 9.5 (8–12) days. Only one case experienced HH recurrence (Type I) on endoscopy and computed tomography but did not have heartburn or dysphagia while on medication. The FSSG scores significantly decreased from 29 (26–35) to 4 (0–7) after surgery (p = 0.0035). Compared with those of four patients who underwent conventional surgeries (hiatal repair with Nissen fundoplication) during the same period, patients undergoing hiatal repair and gastropexy without fundoplication were relatively older (84 [81–96] vs. 74.5 [72–79]), had higher uCCI values (2 [2–5] vs. 1 [0–2]), and higher dysphagia scores (3.5 [3–4] vs. 1 [0–1]). However, there were no differences in the surgical outcomes and postoperative FSSG scores.

**CONCLUSIONS:**

Laparoscopic hiatal repair and gastropexy without fundoplication is feasible in elderly patients with HH type III and high dysphagia scores.

## Abbreviations

FSSG
: frequency scale for the symptoms of GERD
HH
: hiatal hernia
uCCI
: updated Charlson Comorbidity Index

## INTRODUCTION

Laparoscopic surgery for hiatal hernia (HH) has been accepted worldwide, but several problems still exist, including a high recurrence rate, postoperative dysphagia, and reflux.^1)^ The standard surgery for HH has been hiatal repair with fundoplication, which could cause postoperative dysphagia.^2)^ In the aging society, the number of patients presenting with HH type III/IV and severe dysphagia is increasing. Postoperative dysphagia is considered a critical issue in these patients. In recent papers, laparoscopic hiatal repair without fundoplication, which has a lower rate of postoperative dysphagia than hiatal repair with fundoplication, can be an alternative procedure.^3–5)^ Moreover, the SAGES guidelines for the management of hiatal hernia acknowledge that routine fundoplication may not be necessary due to the lack of “high-level evidence to support this practice of routine fundoplication”.^1)^ On the other hand, a recent randomized control study and systematic reviews have shown that gastropexy has been reported to reduce the rate of HH recurrence.^6–8)^ Nevertheless, the indications for hiatal repair without fundoplication should be carefully considered, and postoperative symptoms should be evaluated in detail.

We assessed the feasibility of laparoscopic hiatal repair and gastropexy without fundoplication in 4 patients with HH type III and high dysphagia scores and evaluated postoperative symptoms using the frequency scale for the symptoms of GERD (FSSG).^9,10)^

## CASE PRESENTATION

**Fig. 1:**
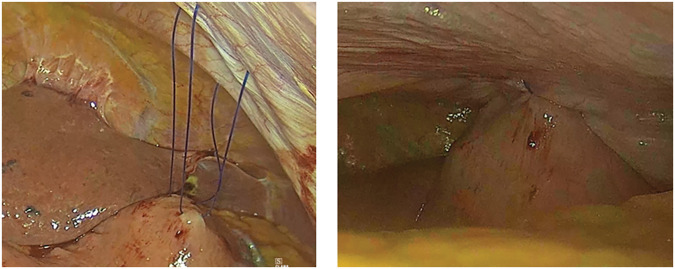
Anterior gastropexy by placing two fixation sutures (2–0 Prolene).

Hiatal repair without fundoplication is performed due to the below criteria: (1) Age >80 years, (2) Hiatal hernia type III, (3) Dysphagia score 3 or 4, (4) LA classification N or M, and (5) Forced expiratory volume in one second >1L. Anterior gastropexy is added in the case that the whole gastric fundus and a part of the short gastric vessels herniate above the diaphragm. At our institution from April 2023 to April 2024, a total of four patients with HH underwent laparoscopic hiatal repair and gastropexy without fundoplication, in which the five-port technique was adopted. The stomach and hernia sac were dissected along the gastric wall, and the abdominal esophagus was mobilized, ensuring that the gastroesophageal junction was not pulled upward. The hiatus was approximated posteriorly with non-absorbable sutures. The gastroesophageal junction was calibrated with a standard flexible endoscope to prevent obstruction. Anterior gastropexy was accomplished by placing two fixation sutures (2–0 Prolene) on the anterior gastric wall adjacent to the greater curvature (**Fig. 1**).

The characteristics and intraoperative results for all four patients are provided in **Table 1**. The median age was 84 (range 81–96) years, and the median value in the updated Charlson Comorbidity Index (uCCI) was 2 (25). All patients had dementia, and Case 4 had congestive heart failure and renal disease in addition. All patients were diagnosed with HH type III and had high dysphagia scores of 3 (n = 2) or 4 (n = 2), but gastrointestinal endoscopy revealed no findings of reflux esophagitis. The median operative time was 196 (157–249) minutes, the median blood loss was 5 (5–15) ml, and the median postoperative hospital stay was 9.5 (8–12) days. One case (Case 3) developed delayed gastric emptying. One month after surgery, fluoroscopy revealed no reflux of contrast into the esophagus in a spine position.

**Table 1:** Patient characteristics and perioperative outcomes

Case no.	Age, years	Sex	BMI, kg/m^2^	uCCI	HH type	Dysphagia score	LA classification^†^	Operative time, min	Blood loss, mL	Postoperative complications	Hospital stay, days
1	81	Female	20.1	2	III	3	Grade N	249	13	None	8
2	82	Female	22.5	2	III	3	Grade N	224	0	None	12
3	86	Female	26.3	2	III	4	Grade N	168	0	Delayed gastric emptying	10
4	96	Female	26.0	5	III	4	Grade N	157	0	None	9

^†^Los Angeles classification

BMI, body mass index; HH, hiatal hernia; uCCI, updated Charlson Comorbidity Index

The postoperative results are shown in **Table 2**. One case (Case 3) was diagnosed with HH recurrence on endoscopy and computed tomography but did not present with heartburn or dysphagia. Cases 1 and 2 had endoscopic findings of reflux esophagitis (grade A) and temporary heartburn, which were treatable with medication.

**Table 2:** Postoperative outcomes

Case no.	HH recurrence	LA classification^†^	Temporary heartburn	Prolonged heartburn	Temporary dysphagia	Prolonged dysphagia
1	No	Grade A	Yes	No	No	No
2	No	Grade A	Yes	No	No	No
3	Yes	Grade N	No	No	No	No
4	No	Grade N	No	No	No	No

^†^Los Angeles classification

HH, hiatal hernia

**Fig. 2:**
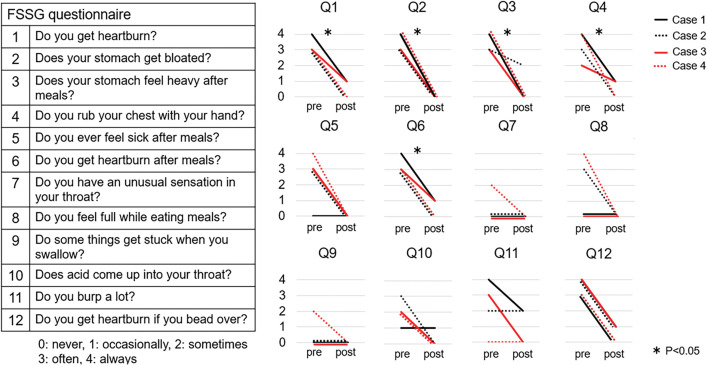
Frequency scale for the symptoms of GERD (FSSG) before and after hiatal repair and gastropexy without fundoplication.

Changes in the FSSG scores before and after surgery are shown in **Fig. 2**. The sum of FSSG scores decreased from 29 (range 26–35) to 4 (0–7) after surgery (p = 0.0035). In questions 1, 2, 3, 4, and 6, there were significant differences when comparing the scores before and after surgery.

Finally, the characteristics and surgical outcomes of this case series were compared with those of 4 patients who underwent conventional surgeries (hiatal repair with Nissen fundoplication) during the same period (**Table 3**). Regarding Nissen fundoplication, we divided the short gastric vessels, created a short and floppy fundic wrap, and fixed the wrap to the abdominal esophagus and diaphragm. Patients undergoing hiatal repair and gastropexy without fundoplication were relatively older (84 [81–96] vs. 74.5 [72–79]), had higher uCCI values (2 [2–5] vs. 1 [0–2]), and higher dysphagia scores (3.5 [3–4] vs. 1 [0–1]), compared with patients who underwent hiatal repair with fundoplication. However, there were no differences in the surgical outcomes and postoperative FSSG scores. One of the patients who underwent hiatal repair with fundoplication developed temporary dysphagia after surgery, which did not require readmission.

**Table 3:** Patient characteristics and surgical outcomes with and without fundoplication

Variables	Fundoplication (n = 4)	No fundoplication (n = 4)
Age, years	74.5 (72–79)	84 (81–96)
Sex, male/female	3/1	0/4
BMI, kg/m^2^	21.4 (19.8–23.7)	24.2 (20.1–26.3)
uCCI	1 (0–2)	2 (2–5)
HH type I/II/III/IV	0/1/3/0	0/0/4/0
Dysphagia score 0/1/2/3/4	1/3/0/0/0	0/0/0/2/2
LA classification^†^ Before surgery Grade N/M/A/B/C/D	3/0/0/0/0/1	4/0/0/0/0/0
FSSG before surgery	19 (13–42)	29 (26–35)
Operative time, min	186 (163–250)	196 (157–249)
Blood loss, ml	9 (5–100)	5 (5–13)
Postoperative complications, n	0	1 (25%)
Hospital stay, days	9.5 (8–12)	9 (9–11)
HH recurrence, n	0	1 (25%)
LA classification^†^ After surgery Grade N/M/A/B/C/D	3/1/0/0/0/0	2/0/2/0/0/0
FSSG after surgery	4.5 (3–7)	4 (0–7)
Temporary heartburn, n	0	2
Prolonged heartburn, n	0	0
Temporary dysphagia, n	1	0
Prolonged dysphagia, n	0	0

^†^Los Angeles classification

BMI, body mass index; FSSG, frequency scale for the symptoms of GERD; HH, hiatal hernia; NA, not applicable; uCCI, upadated Charlson Comorbidity Index

## DISCUSSION

In this case series of four patients, we observed one case of HH recurrence on imaging after surgery without heartburn or dysphagia. Hiatal repair and gastropexy without fundoplication improved FSSG scores similar to conventional surgeries with fundoplication. These findings indicate the feasibility of laparoscopic hiatal repair and gastropexy without fundoplication in patients with HH type III and high dysphagia scores.

In previous systematic reviews, postoperative dysphagia was more common after hiatal repair with fundoplication than hiatal repair without fundoplication.^3)^ In elderly patients with HH, especially type III/IV, postoperative dysphagia could be a concern.^11)^ A case series without fundoplication showed both high dysphagia scores and high FSSG scores for reflux symptoms (questions 1, 4, and 6) preoperatively. However, preoperative endoscopy revealed no findings of reflux esophagitis. After hiatal repair and gastropexy without fundoplication, FSSG scores for reflux symptoms decreased. This means that they possibly considered symptoms of obstruction in the esophagus and stomach as reflux symptoms. Therefore, the surgical priority can be to resolve the dysphagia in elderly patients with HH. We expect that the incidence rate of postoperative dysphagia is lower in hiatal repair without fundoplication than in hiatal repair with fundoplication. Among patients undergoing conventional surgery with fundoplication, one developed postoperative dysphasia, but temporarily. He was discharged on postoperative day 11 and was not readmitted.

In previous papers, surgery without fundoplication showed poor improvement and higher recurrence of gastroesophageal reflux symptoms.^3,12)^ However, a recent large cohort study has reported that routine fundoplication may not be necessary at the time of paraoesophageal HH repair because the fundoplication did not decrease postoperative GERD rates but increased postoperative dysphagia.^5)^ Therefore, the indications for surgery without fundoplication should be carefully considered. In this case series, all patients treated without fundoplication had as good a response on the FSSG as those treated with conventional surgery with fundoplication. This may be because we introduce this surgical procedure to patients with HH type III and high dysphagia scores. On the other hand, postoperative endoscopy revealed findings of reflux esophagitis (grade A) in two cases. There are two reasons for the new onset of reflux esophagitis. First, all patients’ dysphagia scores improved, which was ascribed to the increase in dietary intake. Second, fundoplication may be necessary to completely prevent acid reflux.

A poor response was shown to question 11 on the FSSG (“Do you burp a lot?”) compared with other questions. The reason may be that fundoplication is the only treatment for burping.

Previous papers have reported a higher rate of HH recurrence and reoperation.^3,12)^ In this case series, gastropexy was performed in addition to hiatal repair because gastropexy has been reported to reduce HH recurrence.^6–8,11)^ The rate of recurrence was 25% (1/4), higher than conventional surgery with fundoplication (0%, 4/4). However, reoperation should be indicated if radiological and endoscopic HH recurrence matches symptoms. At least medication can control symptoms of temporary heartburn after surgery as shown in two of our cases.

This case series has several limitations. First, the data were retrospective and from a single institution with potential bias for patient eligibility. Second, a longer follow-up is needed. Finally, due to the small number of enrolled patients, the reliable statistical analysis is difficult to perform. Nevertheless, we believe that hiatal repair without fundoplication is safe because there were no differences in surgical outcomes between hiatal repair with and without fundoplication, though patients without fundoplication showed higher age and higher uCCI. On the other hand, we might overestimate the changes in FSSG scores because symptoms before surgery are more severe in patients without fundoplication.

## CONCLUSIONS

Laparoscopic hiatal repair and gastropexy without fundoplication is feasible in elderly patients with HH type III and high dysphagia scores.

## DECLARATIONS

### Funding

No funding was received toward this work.

### Authors’ contributions

All authors participated in the management of the patient in this case report.

S.M. is the chairperson of our department and supervises the entire process.

All authors read and approved of the final manuscript.

All authors are responsible for the manuscript.

### Availability of data and materials

The data are available on request from the corresponding author, S.U.

### Ethics approval and consent to participate

This work does not require ethical considerations or approval. Informed consent to participate in this study was obtained from the patient.

### Consent for publication

Written informed consent was obtained from the patient for publication of this case report and the accompanying images.

### Competing interests

Drs. Shinya Urakawa, Daishi Yoshimura, Kazuya Sakata, Kimimasa Ikeda, and Satoru Miyazaki have no conflicts of interest or financial ties related to this study.
